# The Gut Microbiome Is Associated With Therapeutic Responses and Toxicities of Neoadjuvant Chemoradiotherapy in Rectal Cancer Patients—A Pilot Study

**DOI:** 10.3389/fcimb.2020.562463

**Published:** 2020-12-09

**Authors:** Wei Shi, Lijun Shen, Wei Zou, Jingwen Wang, Jianing Yang, Yuezhu Wang, Bingdong Liu, Liwei Xie, Ji Zhu, Zhen Zhang

**Affiliations:** ^1^ Department of Radiation Oncology, Fudan University Shanghai Cancer Center, Shanghai, China; ^2^ Department of Oncology, Shanghai Medical College, Shanghai, China; ^3^ Sequencing Department, Chinese National Human Genome Center at Shanghai, Shanghai, China; ^4^ State Key Laboratory of Applied Microbiology Southern China, Guangdong Provincial Key Laboratory of Microbial Culture Collection and Application, Guangdong Open Laboratory of Applied Microbiology, Guangdong Institute of Microbiology, Guangdong Academy of Sciences, Guangzhou, China; ^5^ Zhujiang Hospital, Southern Medical University, Guangzhou, China; ^6^ School of Public Health, Xinxiang Medical College, Xinxiang, China

**Keywords:** gut microbiome, rectal cancer, neoadjuvant chemoradiotherapy, therapeutic responses, toxicities

## Abstract

Responses to neoadjuvant chemoradiotherapy (nCRT) and therapy-related toxicities in rectal cancer vary among patients. To provide the individualized therapeutic option for each patient, predictive markers of therapeutic responses and toxicities are in critical need. We aimed to identify the association of gut microbiome with and its potential predictive value for therapeutic responses and toxicities. In the present study, we collected fecal microbiome samples from patients with rectal cancer at treatment initiation and just after nCRT. Taxonomic profiling *via* 16S ribosomal RNA gene sequencing was performed on all samples. Patients were classified as responders versus non-responders. Patients were grouped into no or mild diarrhea and severe diarrhea. STAMP and high-dimensional class comparisons *via* linear discriminant analysis of effect size (LEfSe) were used to compare the compositional differences between groups. Phylogenetic Investigation of Communities by Reconstruction of Unobserved States (PICRUSt) was utilized to predict differences in metabolic function between groups. Ten patients were classified as responders and 12 patients were classified as non-responders. Fourteen patients experienced no or mild diarrhea and 8 patients experienced severe diarrhea. Several bacteria taxa with significantly different relative abundances before and after nCRT were identified. Similarly, several baseline bacteria taxa and predicted pathways with significantly different relative abundances between responders and non-responders or between patients no or mild diarrhea and severe diarrhea were identified. Specifically, *Shuttleworthia* was identified as enriched in responders and several bacteria taxa in the *Clostridiales* order etc. were identified as enriched in non-responders. Pathways including fatty acid metabolism were predicted to be enriched in responders. In addition, *Bifidobacterium*, *Clostridia*, and *Bacteroides* etc. were identified as enriched in patients with no or mild diarrhea. Pathways including primary bile acid biosynthesis were predicted to be enriched in patients with no or mild diarrhea. Together, the microbiota and pathway markers identified in this study may be utilized to predict the therapeutic responses and therapy-related toxicities of nCRT in patients with rectal cancer. More patient data is needed to verify the current findings and the results of metagenomic, metatranscriptomic, and metabolomic analyses will further mine key biomarkers at the compositional and functional level.

## Introduction

Colorectal cancer is the third most commonly diagnosed cancer in males and the second in females. According to International Agency for Research on Cancer (IARC), 608,700 deaths are estimated to have occurred in 2008, accounting for 8% of the total cancer deaths (Jemal et al., 2011). Approximately, half of the colorectal cancer occurs in rectum. Neoadjuvant chemoradiotherapy (nCRT) has been established as a standard treatment for locally advanced rectal cancer (LARC) (Siegel et al., 2012) and plays an important role in controlling cancer and improving survival. At the same time, nCRT will result in related acute and late toxicities. Acute toxicities, such as diarrhea and myelosuppression, are major impediments to completing the nCRT. Late toxicities, such as fibrosis, greatly affect patients’ quality of life (Greenhalgh et al., 2016).

Responses to nCRT and therapy-related toxicities in rectal cancer vary among patients. To provide the individualized therapeutic option for each patient, predictive markers of therapeutic responses and toxicities are in critical need. It is speculated that factors beyond clinical stage, tumor genomics and germline polymorphism etc (Pezzolo et al., 2015; Dayde et al., 2017) may influence therapeutic responses and toxicities, including host factors such as differential composition of the patients’ gut microbiome (Qin et al., 2010; Arumugam et al., 2011; Koren et al., 2013). Several species of commensal bacteria were shown to play potential roles in colorectal carcinogenesis (Yu et al., 2017; Garrett, 2019; Thomas et al., 2019; Wirbel and Pyl, 2019; Wong and Yu, 2019; Ternes et al., 2020; Yang et al., 2020). More strikingly, commensal bacteria were shown to modulate cancer responses to therapy, including chemotherapy and immunotherapy (Iida et al., 2013; Viaud et al., 2013; Roy and Trinchieri, 2017; Gopalakrishnan and Spencer, 2018; Matson et al., 2018; Routy and Le Chatelier, 2018) and to be correlated with toxicities of chemotherapy or radiotherapy (Husebye et al., 1995; Crawford and Gordon, 2005; Manichanh et al., 2008; Wedlake et al., 2008; Wallace et al., 2010; Nam et al., 2013; Ferreira et al., 2014; Wallace et al., 2015; Wang et al., 2015; Reis Ferreira et al., 2019). By modulating microbiome, the therapeutic responses may be improved (Iida et al., 2013; Sivan et al., 2015; Routy and Le Chatelier, 2018; Matson et al., 2018; Gopalakrishnan and Spencer, 2018) and toxicities may be alleviated (Delia et al., 2002; Delia et al., 2007; Wallace et al., 2010). However, so far, no studies have systematically analyzed the correlation between the gut microbiome and therapeutic responses or toxicities of nCRT in rectal cancer patients (Manichanh et al., 2008; Nam et al., 2013; Wang et al., 2015), except for one fairly recent study, which demonstrated the correlation between pathologic response after preoperative concurrent chemoradiation and gut microbiome composition in rectal cancer patients (Jang, 2020). In addition to the correlation with therapeutic responses, the current study analyzed the correlation of gut microbiome with toxicities of nCRT in rectal cancer patients, which identified the potential microbiota and pathway markers to predict therapeutic responses and toxicities of nCRT in patients with rectal cancer and suggested the possibility to improve therapeutic responses and alleviate toxicities by manipulating microbiome.

## Methods

### Study Design and Subjects

We collected gut (fecal) microbiome samples from 22 inpatients with rectal cancer who were treated with nCRT at treatment initiation and just after nCRT. Patients who received antibiotics, steroids and immunosuppressants within the past 6 months were not included in this study. The stool samples were collected on plastic wrap and transferred to stool sample collection tube. The stool samples were immediately frozen and stored at −80°C before analysis. Patients who did not complete nCRT or were found to have metastatic disease before or at the time of surgery were not included in this study. nCRT is composed of radiotherapy delivered at doses of 50 Gy in 2 Gy daily fractions and concurrent chemotherapy of Capecitabine plus Irinotecan. Radiotherapy was performed according to the institutional protocols. Taxonomic profiling *via* 16S ribosomal RNA gene sequencing was performed on all samples. Using 16S ribosomal RNA gene amplicon sequencing, operational taxonomic units (OTUs) with taxonomic assignment present at different abundance were identified. Basic Local Alignment Search Tool (BLAST) search of the 16S sequences against the National Center for Biotechnology Information (NCBI) database were used to reveal potential species-level identities. To assess the therapeutic responses to nCRT, the 8th edition of the American Joint Committee on Cancer (AJCC) staging system and the College of American Pathologists (CAP) four-point tumor regression grade (TRG) system was used which graded on a scale of 0 (complete response; no viable cancer cells) to 3 (poor response; minimal or no regression, extensive residual cancer) (Siegel et al., 2012). Patients were classified as responders if they have TRG 0-1, ypT0-1, and ypN0 versus non-responders if they have TRG 2-3, ypT2-4, or ypN+. Diarrhea was recorded according to the Common Terminology Criteria for Adverse Events (CTCAE), version 5.0. Patients were grouped into no or mild diarrhea (CTCAE grade 0 or 1) and severe diarrhea (CTCAE grade 2 or higher). The shape and consistency of the stool was recorded according to the Bristol stool scale. The Bristol Stool Chart assessment ranged from type 1 (separate hard lumps) to type 7 (entirely watery) (Lewis and Heaton, 1997). The study was reviewed and approved by the institutional ethics committee of Fudan University Shanghai Cancer Center and written informed consent was obtained from all patients.

### 16S Ribosomal RNA Gene Sequencing

Genomic DNA was extracted using QIAamp DNA Stool Mini Kit (QIAGEN). Amplifications of 16S ribosomal RNA genes V3-4 region were performed with primers 338F and 806R (Huse et al., 2007). The cycling conditions were as follows: denaturation at 95°C for 2 min, 20 cycles of amplification (45 s at 95°C, 30 s at 55°C, and 30 s at 72°C), extension 72°C for 5 min. Three repeat PCR amplifications of each sample were purified with AxyPrep DNA Extraction kit (AXYGEN) and assessed by spectrophotometry (QuantiFluor-ST, Promega). The equimolar amounts of 16S ribosomal RNA PCR amplicons were sequenced on an Illumina MiSeq instrument.

### Bioinformatic and Statistical Analysis

Raw paired FASTQ files were processed using Mothur (version 1.39.5) (Washington et al., 2009). The following exclusion criteria were used for sequence quality control: ambiguous bases, the length shorter than 380bp, chimeric sequence, and contaminant sequence. After data normalization, the SILVA reference database (Quast et al., 2013) (V119) was used as reference for OTU identification under the threshold of 97% similarity. Community richness, evenness and diversity analysis (ACE, Chao, Simpsonenven, Shannon, Simpson, and Good’s coverage) were performed using Mothur. The taxonomic affiliation assignments were based on Ribosomal Database Project (Cole et al., 2009) at default parameter (80% threshold). STAMP was performed to find out the differentially enriched taxa between groups with default parameters and *p*-value < 0.05 (Parks et al., 2014). To further explore the difference, high-dimensional class comparisons *via* linear discriminant analysis of effect size (LEfSe) (Segata et al., 2011) were used. To probe the microbial metabolism and predict metagenome functional content from the marker gene, Phylogenetic Investigation of Communities by Reconstruction of Unobserved States (PICRUSt) was utilized to explore differences of the Kyoto Encyclopedia of Genes and Genomes pathway (KEGG) pathways between groups (Langille et al., 2013).

## Results

### Patients Characteristics and Gut Microbiome

**Figure 1:**
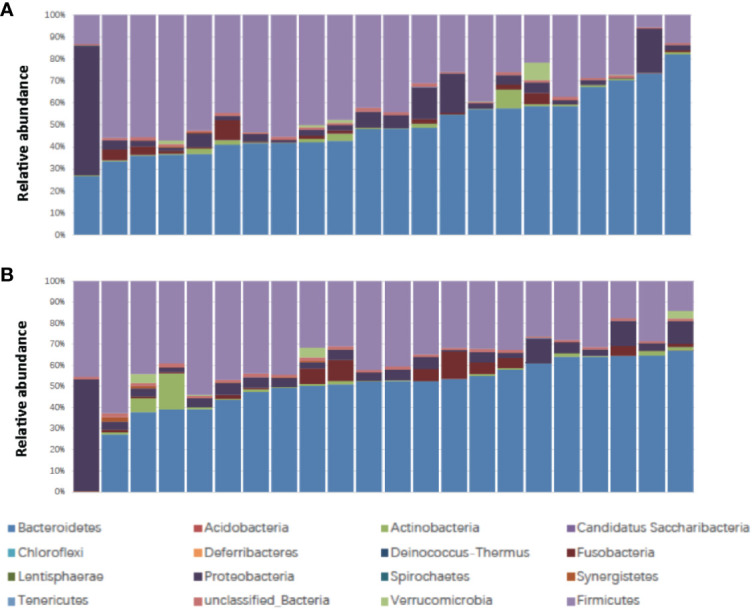
Stacked bar plot of phylogenetic composition of common bacterial taxa at the phylum level (n = 22) **(A)** before nCRT and **(B)** after nCRT. nCRT, neoadjuvant chemoradiotherapy.

The patient characteristics are shown in **Table 1**. There were 22 patients, including 16 male and 6 female patients, aged from 45 to 72 (medium 61). The Body Mass Index (BMI) ranged from 17.6 to 27.0 (medium 22.0) (**Table 1**). Ten patients were classified as responders and 12 patients were classified as non-responders. Fourteen patients experienced no or mild diarrhea and 8 patients experienced severe diarrhea. All of the patients who experienced severe diarrhea (CTCAE grade 2 or higher) presented with Bristol Stool Chart stools of type 6 (fluffy pieces with ragged edges, a mushy stool) or type 7 (entirely watery). Whereas, none of the patients who were grouped into no or mild diarrhea (CTCAE grade 0 or 1) presented with Bristol Stool Chart stools of 7 (entirely watery). No major differences in patient characteristics were observed in responders versus non-responders and patients with no or mild diarrhea versus severe diarrhea (**Table 1**). We first assessed the landscape of the gut microbiome in all available samples in patients with rectal cancer *via* 16S ribosomal RNA gene sequencing, noting that communities were relatively diverse, with a high abundance of *Bacteroidetes* and *Firmicutes* in the gut microbiome (**Figure 1**).

**Table 1:** Baseline characteristics of patients in different groups.

Characteristics	Responders	Non-responders	P^a^	No or mild diarrhea	Severe diarrhea	P^a^
Number	n = 10 (%)	n = 12(%)		n = 14 (%)	n = 8 (%)	
Gender			0.646			1.0
Female	8 (80)	8 (67)		10 (71)	6 (75)	
Male	2 (20)	4 (33)		4 (29)	2 (25)	
Age			0.075			0.7
Medium	59	63		58.5	63	
Range	45–69	54–72		45–72	61–68	
BMI			0.356			0.539
Medium	22.5	21.6		22.5	21.6	
Range	20.8–24.6	17.6–27.0		18.8–25.5	17.6–27.0	
Tumor stage before nCRT			0.455			1.0
I	1 (10)	0 (0)		1 (7)	0 (0)	
III	9 (90)	12 (100)		13 (93)	8 (100)	
Tumor location			0.378			0.649
Low	5 (50)	3 (25)		6 (43)	2 (25)	
High	5 (50)	9 (75)		8 (57)	6 (75)	

nCRT, neoadjuvant chemoradiotherapy. ^a^p-values calculated by Wilcoxon rank sum (age, BMI), Fisher’s exact (all others).

### Impact of nCRT on Gut Microbiome of Rectal Cancer Patients

**Figure 2:**
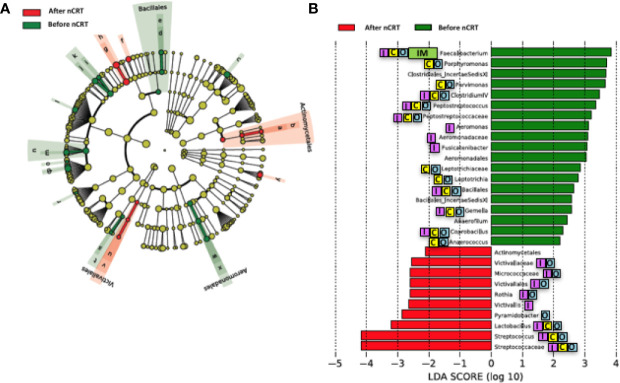
Differences in composition of the gut microbiome before and after nCRT. **(A)** Taxonomic cladogram from LEfSe showing differences in bacterial taxa. Dot size is proportional to the abundance of the taxon. Letters corresponding to the following taxa: a) *Rothia*, b) *Micrococcaceae*, c) *Porphyromonas*, d) *Gemella*, e) *Bacillales Incertae Sedis XI*, f) *Lactobacillus*, g) *Streptococcus*, h) *Streptococcaceae*, i) *Anaerococcus*, j) *Parvimonas*, k) *Clostridiales Incertae Sedis XI*, l) *Fusicatenibacter*, m) *Peptostreptococcus*, n) *Peptostreptococcus*, o) *Anaerofilum*, p) *Clostridium IV*, q) *Faecalibacterium*, r) *Coprobacillus*, s) *Leptotrichia*, t) *Leptotrichiaceae*, u) *Victivallis*, v) *Victivallaceae*, w) *Aeromonas*, x) *Aeromonadaceae*, and y) *Pyramidobacter*. **(B)** LDA scores computed for differentially abundant taxa in the gut microbiome of after nCRT (red) and before nCRT (green). Length indicates effect size associated with a taxon. *p* = 0.05 for the Kruskal-Wallis test; LDA score > 2. nCRT, neoadjuvant chemoradiotherapy. Reported associations: 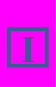 Irritable bowel syndrome; 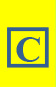 Colorectal cancer; 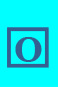 Other cancers; 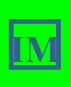 Immunotherapy.

We compared the gut microbiome of before versus after nCRT to investigate the impact of nCRT on gut microbial community in rectal cancer patients. First, we analyzed the richness and diversity in gut microbiome before and after nCRT and no significant differences were noted (**Supplementary Figure 1**), suggesting that nCRT may not introduce dramatic changes to the overall structure of the gut microbial community. Moreover, the bacterial composition and abundance within the gut before and after nCRT were compared and several significantly different bacteria taxa were identified. Eight bacterial taxa including *Prausnitzii* and *Peptostreptococcus* were identified as enriched before nCRT and 3 bacterial taxa including *Splanchnicus* were identified as enriched after nCRT by STAMP (Parks et al., 2014) (**Table 2**). To further explore the differences, high-dimensional class comparisons *via* LEfSe (Segata et al., 2011) was performed, which again demonstrated differentially abundant bacteria in the fecal microbiome, with 19 bacterial taxa including *Faecalibacterium*, *Clostridium IV*, *Porphyromonas*, and *Gemella* identified as enriched in fecal microbiome before nCRT and 10 bacterial taxa identified as enriched after nCRT (**Figure 2**). Among the bacteria taxa identified, *Peptostreptococcus*, *Anaerofilum* and *Fusicatenibacter* were identified as enriched before nCRT, whereas *Micrococcaceae* and *Rothia* were identified as enriched after nCRT by both STAMP and LefSe.

**Table 2:** Comparison of 16S-derived bacteria taxa between before and after nCRT.

Taxon	Abundance before nCRT	Abundance after nCRT	*p*-values	Taxonomy level
*Micrococcaceae* 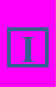 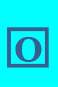	0.00067 ± 0.0021	0.015 ± 0.030	0.044	family
*Rothia* 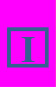 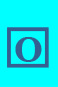	0.00067 ± 0.0021	0.015 ± 0.030	0.044	genus
*Ruminococcus* 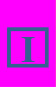 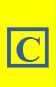 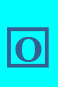	0.88 ± 0.93	0.28 ± 0.44	0.011	genus
*Fusicatenibacter* 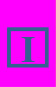	0.38 ± 0.37	0.15 ± 0.18	0.018	genus
*Peptostreptococcus* 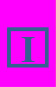 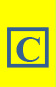 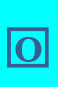	0.47 ± 0.92	0.0094 ± 0.023	0.034	genus
*Anaerofilum*	0.00067 ± 0.0014	0 ± 0	0.042	genus
*Prausnitzii* 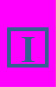 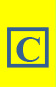 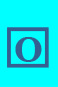	1.9 ± 2.2	0.85 ± 0.96	0.044	species
*Fusicatenibacter saccharivorans* 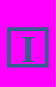	0.32 ± 0.31	0.13 ± 0.15	0.016	species
*Odoribacter splanchnicus* 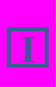	0.045 ± 0.050	0.16 ± 0.23	0.036	species
*Peptostreptococcus/unclassified* (OTU 00087)	0.34 ± 0.65	0.00084 ± 0.019	0.027	species
*Lachnospiraceae/unclassified* (OTU 00192)	0.15 ± 0.28	0.016 ± 0.032	0.049	species

nCRT, Neoadjuvant chemoradiotherapy.

Reported associations: 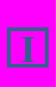 Irritable bowel syndrome; 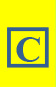 Colorectal cancer; 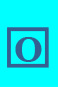 Other cancers.

### Differential Microbiota and Metabolic Functions Between Responders and Non-Responders

**Figure 3:**
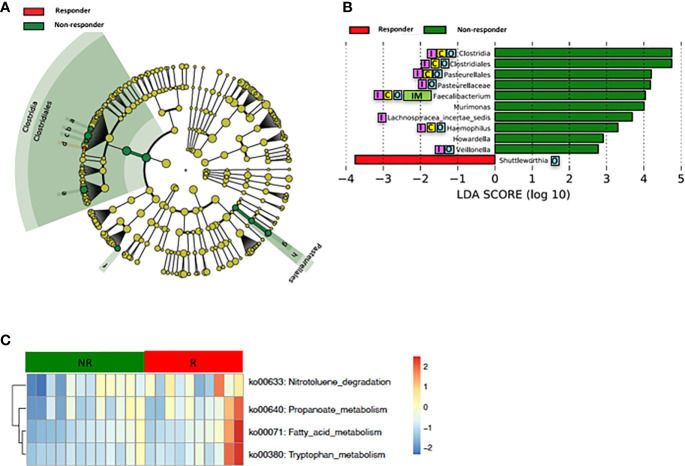
Differences in composition and predicted metagenomic function of the baseline gut microbiome are associated with therapeutic responses to nCRT. **(A)** Taxonomic cladogram from LEfSe showing differences in bacterial taxa. Dot size is proportional to the abundance of the taxon. Letters corresponding to the following taxa: a) *Howardella*, b) *Lachnospiracea incertae sedis*, c) *Murimonas*, d) *Shuttleworthia*, e) *Faecalibacterium*, f) *Veillonella*, g) *Haemophilus*, and h) *Pasteurellaceae*. **(B)** LDA scores computed for differentially abundant taxa in the gut microbiome of responder (red) and non-responder (green). Length indicates effect size associated with a taxon. *p* = 0.05 for the Kruskal-Wallis test; LDA score >2. **(C)** For predicting metagenome function, PICRUSt analysis identified KEGG pathway with significantly different relative abundances, between the two groups. nCRT, neoadjuvant chemoradiotherapy. LEfSe, linear discriminant analysis of effect size. PICRUSt, Phylogenetic Investigation of Communities by Reconstruction of Unobserved States. KEGG, Kyoto Encyclopedia of Genes and Genomes pathway. Reported associations: 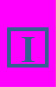 Irritable bowel syndrome; 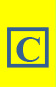 Colorectal cancer; 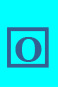 Other cancers; 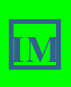 Immunotherapy.

Loss of gut microbial diversity is associated with poor outcomes of cancer therapy, including immunotherapy and cytotoxic or targeted chemotherapy alone or in combination (Gopalakrishnan and Spencer, 2018; Heshiki et al., 2020). Based on these data, we examined the richness and diversity of the baseline gut microbiome in rectal cancer patients treated with nCRT and found that no significant differences were observed between responders and non-responders (responders = 10, non-responders = 12, **Supplementary Figure 2**). Since gut bacterial composition may also influence rectal cancer development (Shen et al., 2010; Azcarate-Peril et al., 2011; Marchesi et al., 2011; Sobhani et al., 2011; Tlaskalova-Hogenova et al., 2011; Chen et al., 2012; Culpepper and Mai, 2012; Garrett, 2019; Thomas et al., 2019; Wirbel and Pyl, 2019) and tumor responses to therapy (Iida et al., 2013; Viaud et al., 2013; Roy and Trinchieri, 2017; Routy and Le Chatelier, 2018; Matson et al., 2018; Gopalakrishnan and Spencer, 2018), we sought to determine if differences existed in the composition and abundance of the baseline gut microbiome between responders and non-responders. To test this, unsupervised hierarchical clustering of OTU abundances within the gut microbiome was then performed without input of response data. Patients were segregated into two distinct community types. Type 1 composed of 7 responders and 7 non-responders whereas type 2 composed of 3 responders and 5 non-responders. No significant difference in the composition of responders and non-responders was identified in different community type (*p* = 0.68) (**Supplementary Figure 3**). We next asked whether relative abundances of specific gut bacteria were associated with the treatment outcome of nCRT. Fifteen bacteria taxa with different relative abundances between responders and non-responders were identified by STAMP (Parks et al., 2014), with all enriched in non-responders including *Parabacteroides merdae* in the *Bacteroidales* order and several bacteria taxa in the *Clostridiales* order (*Ruminococcaceae*/*Faecalibacterium/Prausnitzii*, *Clostridium IV*, *Oscillibacter*, *Romboutsia*, *Blautia*, *Murimonas*, *Lachnospiracea incertae sedis*) (**Table 3**). To further explore the differences, LEfSe (Segata et al., 2011) was performed, which again demonstrated differentially abundant bacteria in the fecal microbiome of responders versus non-responders in response to nCRT, with ten bacterial taxa including several bacteria taxa in the *Clostridiales* order (*Murimonas*, *Faecalibacterium*, *Howardella*, *Lachnospiracea incertae sedis*) and *Haemophilus* enriched in non-responders and 1 bacterial taxa *Shuttleworthia* in the *Clostridiales* order enriched in responders (**Figures 3A, B**). Several bacteria taxa were identified by both STAMP and LefSe as enriched in non-responders, including bacteria taxa in the *Clostridiales* order (*Faecalibacterium*, *Murimonas*, and *Lachnospiracea incertae sedis*). Besides, LEfSe was performed to identify differentially abundant bacteria in the fecal microbiome of before nCRT versus after nCRT in responders or non-responders (**Supplementary Figures 4** and **5**). Next, we attempted to clarify the potential mechanism through which the gut microbiome may influence responses to nCRT, and PICRUSt algorithm (Langille et al., 2013) was performed to assess the functional differences by plotting the differential pathways against KEGG database. Four pathways including fatty acid metabolism (ko00071) were predicted to be enriched in R (Kruskal test *p* < 0.05, **Figure 3C**).

**Table 3:** Comparison of 16S-derived bacteria taxa between responders and non-responders by STAMP.

Taxon	Abundance in responders (%)	Abundance in non-responders (%)	*p*-values	Taxonomy level
*Clostridia* 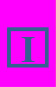 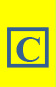 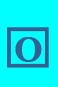	22.83 ± 11.93	33.82 ± 10.85	0.047	class
*Clostridiales* 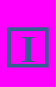 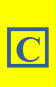 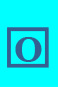	22.83 ± 11.92	33.81 ± 10.85	0.047	order
*Ruminococcaceae* 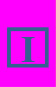 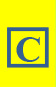 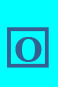	6.08 ± 4.40	12.25 ± 7.10	0.028	family
*Gordonibacter* 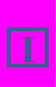	0 ± 0	0.0012 ± 0.0017	0.039	genus
*Murimonas*	0 ± 0	0.0015 ± 0.0018	0.017	genus
*Lachnospiracea incertae sedis* 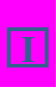	0.80 ± 0.75	1.96 ± 1.34	0.026	genus
*Clostridium IV* 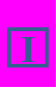 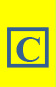 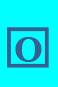	0.26 ± 0.27	1.47 ± 1.61	0.030	genus
*Faecalibacterium* 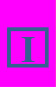 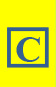 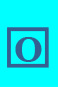 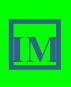	1.10 ± 1.01	3.21 ± 2.91	0.042	genus
*Romboutsia* 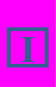	0.048 ± 0.060	0.16 ± 0.15	0.048	genus
*Prausnitzii* 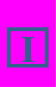 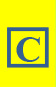 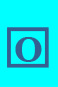 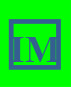	0.93 ± 0.86	2.8 ± 2.5	0.041	species
*Parabacteroides merdae* 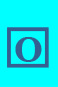	0.097 ± 0.11	0.36 ± 0.37	0.046	species
*Oscillibacter/unclassified* (OTU 00111)	0.059 ± 0.094	0.22 ± 0.21	0.040	species
*Romboutsia/unclassified* (OTU 00153)	0.045 ± 0.056	0.14 ± 0.14	0.049	species
*Blautia/unclassified* (OTU 00178)	0.028 ± 0.049	0.12 ± 0.11	0.030	species
*Ruminococcaceae/unclassified* (OTU 00183)	0 ± 0	0.15 ± 0.20	0.030	species

Reported associations: 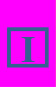 Irritable bowel syndrome; 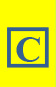 Colorectal cancer; 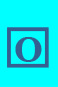 Other cancers; 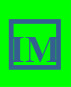 Immunotherapy.

### Differential Microbiota and Metabolic Functions Between No or Mild Diarrhea and Severe Diarrhea

**Figure 4:**
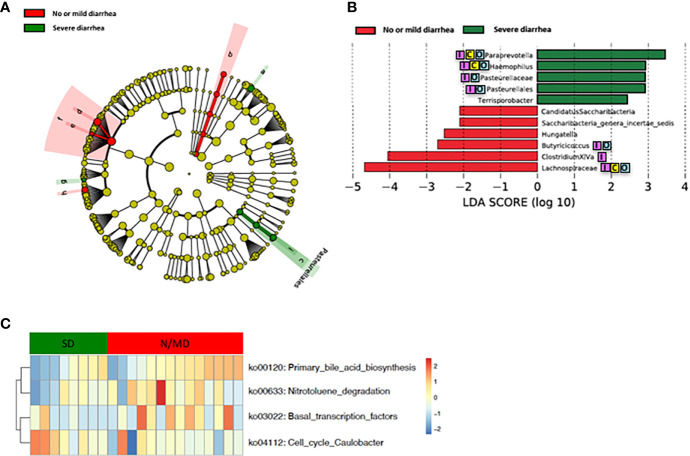
Differences in composition and predicted metagenomic function of the baseline gut microbiome are associated with diarrhea. **(A)** Taxonomic cladogram from LEfSe showing differences in bacterial taxa. Dot size is proportional to the abundance of the taxon. Letters corresponding to the following taxa: a) *Paraprevotella*, b) *Saccharibacteria genera incertae sedis*, c) *Pasteurellaceae*, d) *Clostridium XlVa*, e) *Hungatella*, f) *Lachnospiraceae*, g) *Terrisporobacter*, h) *Butyricicoccus*, and i) *Haemophilus*. **(B)** LDA scores computed for differentially abundant taxa in the gut microbiome of no or mild diarrhea (red) and severe diarrhea (green). Length indicates effect size associated with a taxon. *p* = 0.05 for the Kruskal-Wallis test; LDA score >2. **(C)** For predicting metagenome function, PICRUSt analysis identified KEGG pathway with significantly different relative abundances, between the two groups. LEfSe: linear discriminant analysis of effect size. PICRUSt: Phylogenetic Investigation of Communities by Reconstruction of Unobserved States. KEGG: Kyoto Encyclopedia of Genes and Genomes pathway. Reported associations: 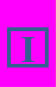 Irritable bowel syndrome; 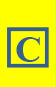 Colorectal cancer; 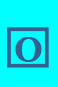 Other cancers.

Loss of microbial diversity and bacterial composition/abundances are associated with therapy-related toxicities of cancer (Husebye et al., 1995; Crawford and Gordon, 2005; Manichanh et al., 2008; Wedlake et al., 2008; Wallace et al., 2010; Nam et al., 2013; Ferreira et al., 2014; Wallace et al., 2015; Wang et al., 2015; Reis Ferreira et al., 2019). Therefore, we examined the richness and diversity of the gut microbiome in rectal cancer patients treated with nCRT and found that no significant differences were observed between patients with no or mild diarrhea and severe diarrhea (no or mild diarrhea = 14, severe diarrhea = 8, **Supplementary Figure 6**). In addition, we sought to determine if differences existed in the composition and abundances of the gut microbiome of patients with no or mild diarrhea and severe diarrhea. To test this, unsupervised hierarchical clustering of OTU abundances within the gut microbiome was performed without input of toxicity data. Patients were segregated into two distinct community types. Type 1 composed of 10 patients with no or mild diarrhea and 4 patients with severe diarrhea whereas type 2 composed of 4 patients with no or mild diarrhea and 4 patients with severe diarrhea. No significant difference in the composition of no or mild diarrhea and severe diarrhea was identified in different community type (*p* = 0.39, **Supplementary Figure 7**). We next asked whether relative abundances of specific gut bacteria were associated with the severity of diarrhea related with nCRT. Several bacteria taxa with different relative abundances between no or mild diarrhea and severe diarrhea were identified by STAMP, with all 16 including *Bifidobacterium* and several bacteria taxa in the *Clostridia* class and in the *Bacteroides* genus enriched in patients with no or mild diarrhea (**Table 4**). To further explore the differences, LEfSe (Segata et al., 2011) was performed, which again demonstrated differentially abundant bacteria in the fecal microbiome of patients with no or mild diarrhea versus those with severe diarrhea, with 6 bacterial taxa enriched in patients with no or mild diarrhea and 5 bacterial taxa including *Pasteurellaceae/Haemophilus* enriched in patients with severe diarrhea (**Figures 4A, B**). Among bacteria taxa identified, several bacteria taxa in the *Clostridia* class (*Butyricicoccus*, *Clostridium XlVa*, and *Hungatella*) were identified by both STAMP and LefSe as enriched in patients with no or mild diarrhea. Besides, LEfSe was performed to identify differentially abundant bacteria in the fecal microbiome of before nCRT versus after nCRT in patients with no or mild diarrhea or those with severe diarrhea (**Supplementary Figures 8** and **9**). Next, we attempted to elucidate the potential mechanism through which the gut microbiome may influence the severity of diarrhea related with nCRT, and PICRUSt algorithm (Langille et al., 2013) was performed to assess the functional differences by plotting the differential pathways against KEGG database. Three pathways including primary bile acid biosynthesis (ko00120) were predicted to be enriched in patients with no or mild diarrhea and one pathway (cell cycle Caulobacter, ko04112) was predicted to be enriched in patients with severe diarrhea (Kruskal test *p* < 0.05, **Figure 4C**).

**Table 4:** Comparison of 16S-derived bacteria taxa between patients with no or mild diarrhea and those with severe diarrhea by STAMP.

Taxon	Abundance in no or mild diarrhea (%)	Abundance in severe diarrhea (%)	*p*-values	Taxonomy level
*Butyricicoccus* 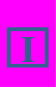 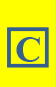 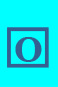	0.17 ± 0.084	0.078 ± 0.041	0.0046	genus
*Clostridium XlVa* 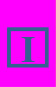	2.9 ± 2.1	0.88 ± 0.52	0.064	genus
*Hungatella*	0.0037 ± 0.005	0 ± 0	0.025	genus
*Bacteroides vulgatus* 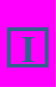	7.8 ± 7.3	2.5 ± 2.8	0.033	species
*Bifidobacterium/unclassified* (OTU 00022)	0.73 ± 1.0	0.06 ± 0.10	0.039	species
*Bacteroides xylanisolvens*	0.84 ± 1.2	0.084 ± 0.056	0.041	species
*Roseburia/unclassified* (OTU 00038)	0.76 ± 0.77	0.19 ± 0.35	0.036	species
*Bacteroides/unclassified* (OTU 00059)	0.46 ± 0.46	0.070 ± 0.087	0.011	species
*Bacteroides/unclassified* (OTU 00071)	0.29 ± 0.27	0.070 ± 0.10	0.016	species
*Bacteroides/unclassified* (OTU 00077)	0.27 ± 0.35	0.052 ± 0.084	0.049	species
*Flavonifractor plautii* 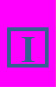	0.12 ± 0.14	0.018 ± 0.024	0.019	species
*Bacteroides/unclassified* (OTU 00110)	0.16 ± 0.17	0.020 ± 0.018	0.012	species
*Clostridiales/unclassified* (OTU 00114)	0.14 ± 0.18	0.026 ± 0.022	0.039	species
*Blautia/unclassified* (OTU 00190)	0.062 ± 0.052	0.019 ± 0.015	0.013	species
*Lachnospiraceae/unclassified* (OTU 00192)	0.22 ± 0.33	0.013 ± 0.016	0.040	species
*Bacteroides/unclassified* (OTU 00194)	0.092 ± 0.10	0.015 ± 0.017	0.017	species

Reported associations: 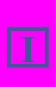 Irritable bowel syndrome; 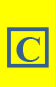 Colorectal cancer; 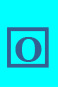 Other cancers.

## Discussion

Our study demonstrated that no significant differences in the richness and diversity in gut microbiome before and after nCRT were noted, indicating that nCRT may not introduce remarkable changes to the overall structure of the gut microbial community. It is possible that nCRT may either act as a selective pressure for more resistant bacteria taxa, or that bacteria taxa which are sensitive to nCRT may acquire mutations during the course of treatment, that enable them to evade treatment-induced death. Several bacteria taxa with significantly different abundances before and after nCRT were identified by STAMP (Parks et al., 2014) and LefSe (Segata et al., 2011) (**Figure 2** and **Table 2**). The changes in gut microbiome induced by chemotherapy and radiotherapy may contribute to the development of mucositis, particularly diarrhea (Touchefeu et al., 2014). Several bacteria taxa identified were shown to be enriched in colorectal cancer patients (Ternes et al., 2020), including *Porphyromonas*, *Parvimonas*, *Gemella* identified by LefSe (**Figure 2**), *Peptostreptococcus* identified by STAMP as enriched before nCRT (**Table 2**), and *Splanchnicus* identified as enriched after nCRT by STAMP (**Table 3**). *Prausnitzii*, as identified by STAMP as enriched before nCRT, was shown to be reduced in colorectal cancer patients (Burns et al., 2015; Nakatsu et al., 2015).

Our study showed that gut microbiota was not only affected by nCRT, but more importantly, that baseline gut microbial features may serve as a predictive tool to identify patients who are more likely to benefit from nCRT and less likely to develop diarrhea. The correlations between therapeutic efficacy and gut microbiome were analyzed. Several bacteria taxa with significantly different abundances between responders and non-responders were identified (**Figures 3A, B** and **Table 4**), although no significant differences in the richness/diversity in bacterial microbiome as well as microbial profile were noted (**Supplementary Figures 2** and **3**). Our finding that member of the *Bacteroidales* order including *Parabacteroides merdae* is enriched in non-responders, was consistent with the enrichment of *Bacteroidales* order in non-responders of melanoma patients who received anti–PD-1 immunotherapy (Gopalakrishnan and Spencer, 2018) and the enrichment in non-responders of rectal cancer patients who received preoperative concurrent chemoradiation (Jang, 2020). It is unexpected and needs further validation that *Faecalibacterium/Prausnitzii* was identified as enriched in non-responders compared to responders (**Figure 3**) and also enriched in non-responders before nCRT compared to after nCRT (**Supplementary Figure 5**), since *Faecalibacterium/Prausnitzii* was suggested to be positively correlated with CD8+ T cell infiltrate in the tumor and favorable responses to anti–PD-1 immunotherapy (Gopalakrishnan and Spencer, 2018). Besides, several bacteria taxa including *Clostridium IV* and *Haemophilus*, which were identified as enriched in non-responders, were previously shown to be associated with colorectal cancer patients (Yu et al., 2017; Ternes et al., 2020). Other than the compositional differences of gut microbiota between responders and non-responders, PICRUSt algorithm was utilized to predict functional differences. We identified pathways such as fatty acid metabolism (ko00071), nitrotoluene degradation (ko00633), propanoate metabolism (ko00640), and tryptophan metabolism (ko00380) that were predicted to be enriched in responders (Kruskal test *p* < 0.05, **Figure 3C**). Fatty acid metabolism was shown to be enriched in gut microbiota of responders versus non-responders of melanoma patients who received anti–PD-1 immunotherapy (Gopalakrishnan and Spencer, 2018). Besides, short chain fatty acids (SCFAs), including propanoate, was shown to mediate a number of important functions for the host, including inhibiting proliferation and inducing apoptosis of colorectal cancer cells (DesulfovibrionaceaeLouis et al., 2014; Honda and Littman, 2016; Sonnenburg and Backhed, 2016; Blacher et al., 2017). Our data and the above-mentioned previous data indicate that enhanced activity of fatty acid metabolism and propanoate metabolism may be associated with improved therapeutic efficacy of anti-tumor therapy. Whether boosting the activity of fatty acid and propanoate metabolism etc. may improve therapeutic efficacy requires further investigation. It is unexpected that tryptophan metabolism was predicted to be enriched in responders, since tryptophan metabolism was reported to promote tumor progression (Platten et al., 2019). These results should be interpreted cautiously due to the limitation of predictive nature of PICRUSt (Langille et al., 2013). Similarly, in our study, several baseline bacteria taxa with significantly different abundances were identified between patients with no or mild diarrhea and those with severe diarrhea (**Figures 4A, B** and **Tables 3** and **4**), although no significant differences in the richness/diversity in gut microbiome as well as microbial profile were noted (**Supplementary Figures 4** and **5**). Although our results could not identify causal relationship between any of those bacteria taxa with protection from therapy-related diarrhea, it is worth noting that several bacteria taxa of the *Clostridia* class including *Clostridium XlVa*, which was shown to help expansion and differentiation of regulatory T cell and attenuate colitis and allergic diarrhea (Atarashi et al., 2013), was significantly more abundant in patients with no or mild diarrhea. Similarly, it is of interest that *Bifidobacterium* was identified to be enriched in patients with no or mild diarrhea compared to those with severe diarrhea by STAMP. *Bifidobacterium* is one of the most studied bacteria taxa that could potentially alleviate diarrhea caused by infection and antibiotic, etc. (Wilkins and Sequoia, 2017). Strains of *Bifidobacterium* were included as components of probiotic products, which demonstrated potential to prevent gastrointestinal toxicity related with chemotherapy and radiotherapy (Ciorba et al., 2015). It has been shown that *Bifidobacterium infantis* could drive the generation and function of regulatory T cells which control excessive NF-kB activation (O’Mahony et al., 2008). Similarly, our finding that members of the *Bacteroides* genus including *Bacteroides vulgatus* and several unclassified species of *Bacteroides* are enriched in patients with no or mild diarrhea, was consistent with a proposed role of these commensal bacteria to limit inflammation by stimulating T regulatory cell differentiation (Faith et al., 2014; Dubin et al., 2016). Other than the compositional differences of gut microbiota between responders and non-responders, PICRUSt algorithm was utilized to predict functional differences. We identified pathways such as basal transcription factors (ko03022), primary bile acid biosynthesis (ko00120) and nitrotoluene degradation (ko00633) that were predicted to be enriched in the patients with no or mild diarrhea (Kruskal test *p* < 0.05), while cell cycle *Caulobacter* (ko04112) associated pathways were enriched in the patients with severe diarrhea (Kruskal test *p* < 0.05) (**Figure 4C**). It is worth noting that the severity of diarrhea for some patients after nCRT might be potentially underestimated since the frequency of bowel movements falls as the tumor responded to nCRT and shrinked.

In summary, the current study identified the potential microbiota and pathway markers to predict the therapeutic responses and toxicities of nCRT in patients with rectal cancer. In addition, the current study has indicated that gut microbiota and their metabolites maybe a mediator affecting therapeutic responses and toxicities and shed lights on the potential mechanisms, which provides the theoretic basis to improve the efficacy and reduce toxicity by manipulating microbiota and their specific pathways. However, limited by the small patient cohort of the current study and utilization of only16S ribosomal RNA gene sequencing, more patient data is needed to verify the current findings and the application of metagenomic, metatranscriptomic, and metabolomic analyses will further mine key biomarkers at the compositional and functional level. Moreover, limited by the correlation nature of the current study, future in-depth mechanistic studies are needed to establish the causal relationship between specific bacteria taxa and therapeutic efficacy and toxicity.

## Conclusion

Our study demonstrated that nCRT resulted in significant changes in the relative abundances of several bacteria taxa (**Figure 2** and **Table 3**), even though no significant differences in the richness and diversity in bacterial microbiome before and after nCRT were noted (**Supplementary Figure 1**). Similarly, several bacteria taxa and predicted KEGG pathways with significantly different abundances between responders and non-responders or between patients with no or mild diarrhea and those with severe diarrhea were identified (**Figures 3** and **4** and **Tables 3** and **4**), though no significant differences in the richness and diversity in bacterial microbiome as well as microbial profile were noted (**Supplementary Figures 2–5**). Specifically, *Shuttleworthia* was identified as enriched in responders and several bacteria taxa in the *Clostridiales* order etc. were identified as enriched in non-responders. Pathways including fatty acid metabolism were predicted to be enriched in responders. In addition, *Bifidobacterium*, *Clostridia*, and *Bacteroides* etc. were identified as enriched patients with no or mild diarrhea. Pathways including primary bile acid biosynthesis were predicted to be enriched in patients with no or mild diarrhea. The microbiota and pathway markers revealed in this study may be used to predict the therapeutic responses and toxicities of nCRT in patients with rectal cancer. More patient data is needed to verify the current findings and future in-depth mechanistic studies are needed to establish the causal relationship between specific bacteria taxa and therapeutic efficacy and toxicity.

## Data Availability Statement

All the sequencing results are deposited in NODE (The National Omics Data Encyclopedia) (https://www.biosino.org/node) under the project ID: OEP001256.

## Ethics Statement

The studies involving human participants were reviewed and approved by Fudan University Shanghai Cancer Center. The patients/participants provided their written informed consent to participate in this study. Written informed consent was obtained from the individual(s) for the publication of any potentially identifiable images or data included in this article.

## Author Contributions

WS and ZZ were involved in study conception and study design. LS, WZ, JZ, and ZZ enrolled the subjects. WS, LS, WZ, JY collected the stool samples. LS, WZ, and JW participated in patient follow-ups and recorded patients’ information. YW performed the bioinformatics analysis of the sequencing data. BL and LX performed PICRUSt analysis. The manuscript was prepared by WS, LS, and ZZ. All authors contributed to the article and approved the submitted version.

## Funding

This work was supported by the Shanghai Pujiang Talent Program (Grant Number: 16PJ1402000), National Natural Science Foundation of China (No. 81773357), Shanghai ACA Foundation (SACACY19B04) and GDAS' Project of Science and Technology Development (No. 2018GDASCX-0102).

## Conflict of Interest

The authors declare that the research was conducted in the absence of any commercial or financial relationships that could be construed as a potential conflict of interest.
