# Supplementary material for: The Gut Microbiome Is Associated With Therapeutic Responses and Toxicities of Neoadjuvant Chemoradiotherapy in Rectal Cancer Patients—A Pilot Study
Source: Front Cell Infect Microbiol. 2020 Dec 9;10:562463. doi: 10.3389/fcimb.2020.562463 (PMC7756020; doi:10.3389/fcimb.2020.562463)
Supplement: Supplementary file 1 [file DataSheet_1.docx]

The gut microbiome is associated with therapeutic responses and toxicities of neoadjuvant chemoradiotherapy in rectal cancer patients

Wei Shi^1,2†^*, Lijun Shen^1,2†^ , Wei Zou^1,2†^, Jingwen Wang^1,2^, Jianing Yang^1,2^, Yuezhu Wang^3^, Bingdong Liu^4^, Liwei Xie^4,5,6^, Ji Zhu^1,2^, Zhen Zhang^1,2^*

^1^ Department of Radiation Oncology, Fudan University Shanghai Cancer Center, Shanghai, China; lijunshen@fudan.edu.cn (L.S.); daychanging@163.com (W.Z.); jingwenwang12@fudan.edu.cn (J.W.); fsyjn888@163.com (J.Y.); leo.zhu@126.com (J.Z.)

^2^ Department of Oncology, Shanghai Medical College, Shanghai, China;

^3^ Chinese National Human Genome Center at Shanghai, Shanghai, China; wangyuezhu@chgc.sh.cn (Y.W.)

^4^ State Key Laboratory of Applied Microbiology Southern China, Guangdong Provincial Key Laboratory of Microbial Culture Collection and Application, Guangdong Open Laboratory of Applied Microbiology, Guangdong Institute of Microbiology, Guangdong Academy of Sciences, Guangzhou, China; liubingdong@stu2016.jnu.edu.cn (B.L.); xielw@gdim.cn (L.X.)

^5^ Zhujiang Hospital, Southern Medical University, Guangzhou, China

^6^ School of public health, Xinxiang Medical College, Xinxiang, China

^*^ Correspondence: wei.shi118@foxmail.com (W.S.); zhenzhang6@gmail.com (Z.Z.)

^†^ These authors contributed equally to this work as co-first author.

Supplementary Materials:


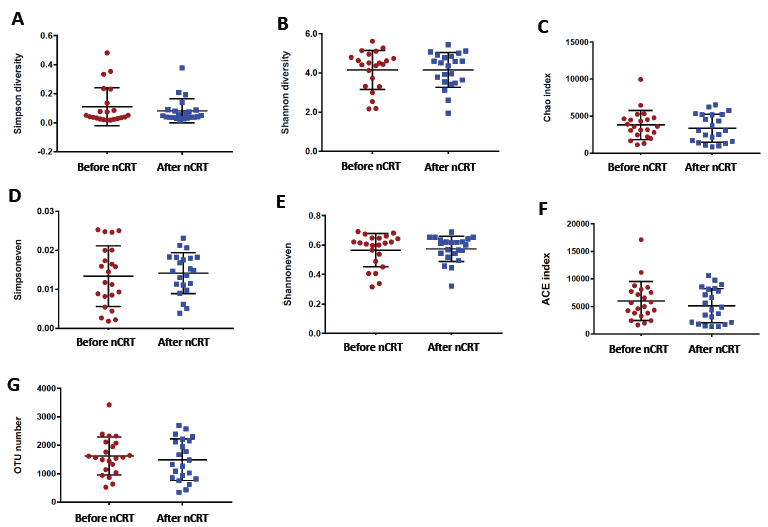


**Supplementary Figure 1** No differences are observed in the diversity and OTUs of the microbiome before and after nCRT. Comparison of alpha diversity scores of before and after CRT using the (A) Simpson (*p*=0.36), (B) Shannon (*p*=0.97), (C) Chao (*p*=0.35) and (D) Simpsoneven (*p*=0.63), (E) Shannoneven (*p*=0.75), (F) ACE (*p*=0.29) indices, (G) OTUs (*p*=0.42) by the t-test. nCRT: neoadjuvant chemoradiotherapy. OTU: operational taxonomic unit.


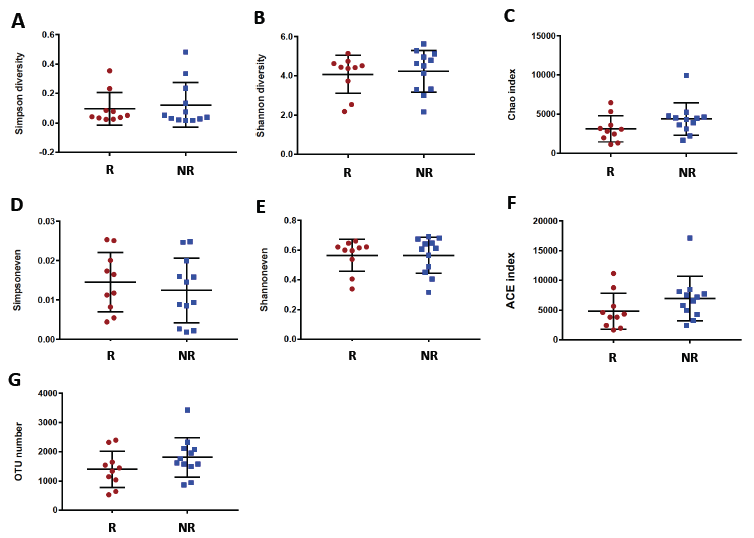


**Supplementary Figure 2** No differences are observed in the richness and diversity in gut microbiome before nCRT between R and NR. Comparison of diversity and richness of gut microbiome between R and NR using the (A) Simpson (*p*=0.65), (B) Shannon (*p*=0.71), (C) Chao (*p*=0.14) and (D) Simpsoneven (*p*=0.54), (E) Shannoneven (*p*=0.98), (F) ACE (*p*=0.16) indices, (G) OTUs (*p*=0.16) by the t-test. R: responder. NR: non-responder. nCRT: neoadjuvant chemoradiotherapy. OTU: operational taxonomic unit.


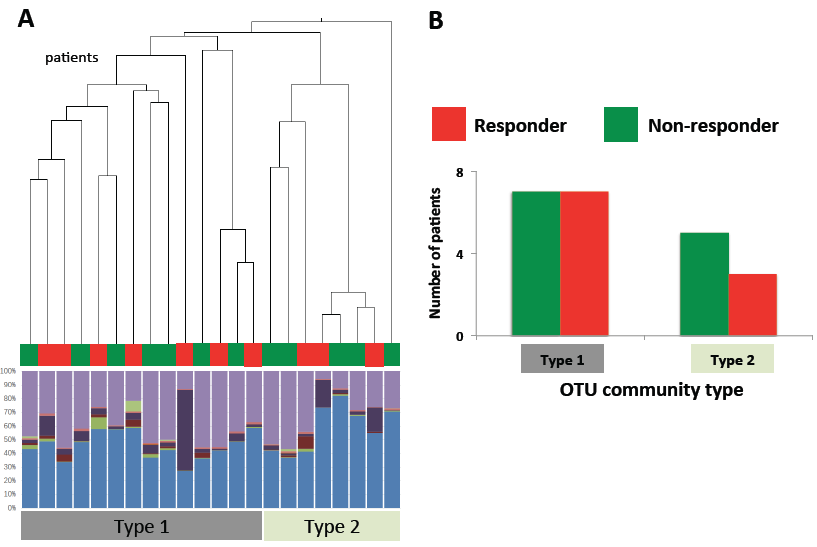


**Supplementary Figure 3** Abundance of OTUs within gut microbiome in R and NR. (A) Top: unsupervised hierarchical clustering by complete linkage of Euclidean distances of OTU abundances (n=22). Bottom: Stacked bar plot of relative abundances at the phylem level by OTU community-type. (B) Association of OTU community types with response to nCRT by Fisher’s exact test (*p*=0.68). OTU community type 1 (gray, n=14: R=7, NR=7); OTU community type 2 (pale green, n=8: R=3, NR=5). Red bars indicate responders, whereas green bars indicate non-responders. nCRT: neoadjuvant chemoradiotherapy. OTU: operational taxonomic unit.

**Supplementary Figure 4** Differences in composition of the gut microbiome in responders before and after nCRT. (A) Taxonomic cladogram from LEfSe showing differences in bacterial taxa. Dot size is proportional to the abundance of the taxon. Letters corresponding to the following taxa: a) *Gemella,* b) *Bacillales Incertae Sedis XI*, c) *Anaerococcus*, d) *Parvimonas*, e) *Clostridiales_Incertae Sedis XI*, f) *Eisenbergiella*, g) *Peptostreptococcus*, h) *Clostridium IV*, i) *Aeromonas*, j) *Aeromonadaceae*, k) *Pyramidobacter*. (B) LDA scores computed for differentially abundant taxa in the gut microbiome of after nCRT (red) and before nCRT (green). Length indicates effect size associated with a taxon. *p*=0.05 for the Kruskal-Wallis test; LDA score >2. nCRT: neoadjuvant chemoradiotherapy.

**Supplementary Figure 5** Differences in composition of the gut microbiome in non-responders before and after nCRT. (A) Taxonomic cladogram from LEfSe showing differences in bacterial taxa. Dot size is proportional to the abundance of the taxon. Letters corresponding to the following taxa: a) *Rothia*, b) *Micrococcaceae*, c) *Porphyromonas*, d) *Gemella*, e) *Bacillales_Incertae Sedis XI*, f) *Staphylococcus*, g) *Staphylococcaceae*, h) *Parvimonas*, i) *Clostridiales Incertae Sedis XI*, j) *Fusicatenibacter*, k) *Roseburia*, l) *Peptostreptococcus*, m) *Faecalibacterium*, n) *Ruminococcus*, o) *Coprobacillus*. (B) LDA scores computed for differentially abundant taxa in the gut microbiome of after nCRT (red) and before nCRT (green). Length indicates effect size associated with a taxon. *p*=0.05 for the Kruskal-Wallis test; LDA score >2. nCRT: neoadjuvant chemoradiotherapy.


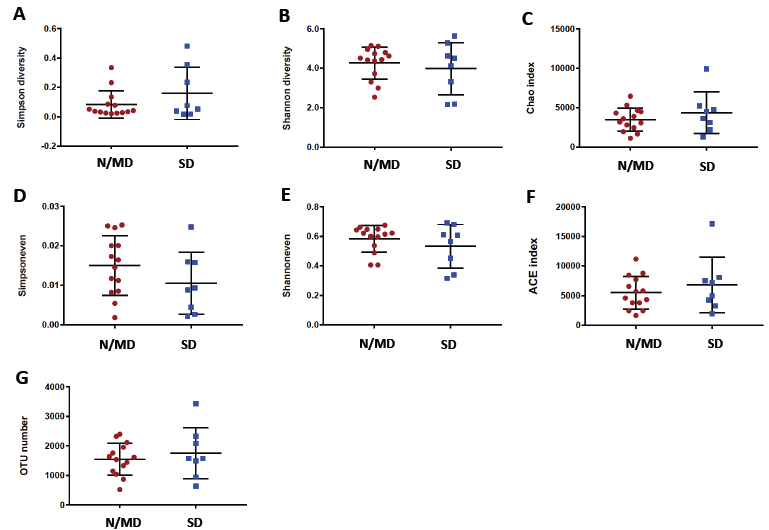


**Supplementary Figure 6** No differences are observed in the richness and diversity of gut microbiome before nCRT between N/MD and SD. Comparison of richness and diversity of gut microbiome before nCRT using the (A) Simpson (*p*=0.20), (B) Shannon (*p*=0.54), (C) Chao (*p*=0.34) and (D) Simpsoneven (*p*=0.20), (E) Shannoneven (*p*=0.33), (F) ACE (*p*=0.42) indices, (G) OTUs (*p*=0.50) by the t-test. nCRT: neoadjuvant chemoradiotherapy. N/MD: no or mild diarrhea. SD: severe diarrhea. OTU: operational taxonomic unit.


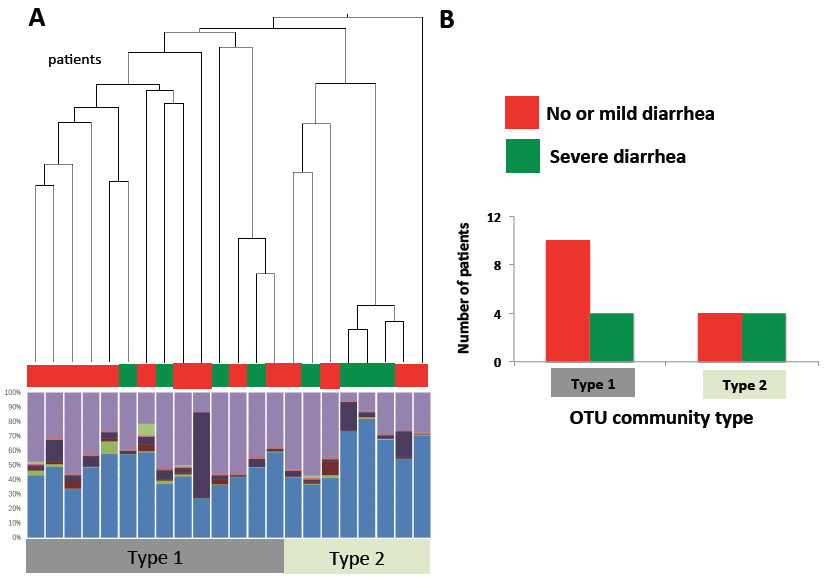


**Supplementary Figure 7** Abundance of OTUs within gut microbiome in N/MD and SD. (A) Top: unsupervised hierarchical clustering by complete linkage of Euclidean distances of OTU abundances (n=22). Bottom: Stacked bar plot of relative abundances at the phylem level by OTU community-type. (B) Association of OTU community types with diarrhea by Fisher’s exact test (*p*=0.39). OTU community type 1 (gray, n=14: N/MD=10, SD=4); OTU community type 2 (pale green, n=8: N/MD=4, SD=4). Red bars indicate N/MD, whereas green bars indicate SD. N/MD: no or mild diarrhea. SD: severe diarrhea. OTU: operational taxonomic unit.

**Supplementary Figure 8** Differences in composition of the gut microbiome in patients with no or mild diarrhea before and after nCRT. (A) Taxonomic cladogram from LEfSe showing differences in bacterial taxa. Dot size is proportional to the abundance of the taxon. Letters corresponding to the following taxa: a) *Porphyromonas*, b) *Gemella*, c) *Bacillales_Incertae Sedis XI*, d) *Enterococcus*, e) *Enterococcaceae*, f) *Anaerococcus*, g) *Parvimonas*, h) *Clostridiales Incertae Sedis XI*, i) *Clostridium XlVa*, j) *Eisenbergiella*, k) *Peptostreptococcus*, l) *Peptostreptococcaceae*, m) *Clostridium IV*, n) *Coprobacillus*, o) *Erysipelotrichia*, p) *Leptotrichiaceae*, q) *Aeromonas*, r) *Aeromonadaceae*, s) *Pyramidobacter*. (B) LDA scores computed for differentially abundant taxa in the gut microbiome of after nCRT (red) and before nCRT (green). Length indicates effect size associated with a taxon. *p*=0.05 for the Kruskal-Wallis test; LDA score >2. nCRT: neoadjuvant chemoradiotherapy.

**Supplementary Figure 9** Differences in composition of the gut microbiome in patients with severe diarrhea before and after nCRT. (A) Taxonomic cladogram from LEfSe showing differences in bacterial taxa. Dot size is proportional to the abundance of the taxon. Letters corresponding to the following taxa: a) *Atopobium*, b) *Gemella*, c) *Bacillales Incertae Sedis XI*, d) *Parvimonas*, e) *Clostridiales Incertae Sedis XI*. (B) LDA scores computed for differentially abundant taxa in the gut microbiome of after nCRT (red) and before nCRT (green). Length indicates effect size associated with a taxon. *p*=0.05 for the Kruskal-Wallis test; LDA score >2. nCRT: neoadjuvant chemoradiotherapy.
